# Identifying Responsive Modules by Mathematical Programming: An Application to Budding Yeast Cell Cycle

**DOI:** 10.1371/journal.pone.0041854

**Published:** 2012-07-25

**Authors:** Zhenshu Wen, Zhi-Ping Liu, Yiqing Yan, Guanying Piao, Zhengrong Liu, Jiarui Wu, Luonan Chen

**Affiliations:** 1 Key Laboratory of Systems Biology, SIBS-Novo Nordisk Translational Research Centre for PreDiabetes, Shanghai Institutes for Biological Sciences, Chinese Academy of Sciences, Shanghai, China; 2 Department of Mathematical Sciences, South China University of Technology, Guangzhou, China; 3 Hefei National Laboratory for Physical Sciences at Microscale and School of Life Sciences, University of Science and Technology of China, Hefei, China; University of Georgia, United States of America

## Abstract

High-throughput biological data offer an unprecedented opportunity to fully characterize biological processes. However, how to extract meaningful biological information from these datasets is a significant challenge. Recently, pathway-based analysis has gained much progress in identifying biomarkers for some phenotypes. Nevertheless, these so-called pathway-based methods are mainly individual-gene-based or molecule-complex-based analyses. In this paper, we developed a novel module-based method to reveal causal or dependent relations between network modules and biological phenotypes by integrating both gene expression data and protein-protein interaction network. Specifically, we first formulated the identification problem of the responsive modules underlying biological phenotypes as a mathematical programming model by exploiting phenotype difference, which can also be viewed as a multi-classification problem. Then, we applied it to study cell-cycle process of budding yeast from microarray data based on our biological experiments, and identified important phenotype- and transition-based responsive modules for different stages of cell-cycle process. The resulting responsive modules provide new insight into the regulation mechanisms of cell-cycle process from a network viewpoint. Moreover, the identification of transition modules provides a new way to study dynamical processes at a functional module level. In particular, we found that the dysfunction of a well-known module and two new modules may directly result in cell cycle arresting at S phase. In addition to our biological experiments, the identified responsive modules were also validated by two independent datasets on budding yeast cell cycle.

## Introduction

High-throughput biological technologies allow the simultaneous measurement of the expression of thousands of genes or proteins, which offers an unprecedented opportunity to fully characterize biological processes [1]. Nevertheless, extracting a comprehensive overview from the huge amount of information is a significant challenge [2]. During the last decade, high-throughput analysis mainly focused on dissecting the individual genes responsible for specific phenotypes, and some biomarkers for human diseases have successfully been identified through analysis of genome-wide expression profiles [3], [4], [5], [6]. However, it is well accepted that genes or proteins within a cell do not function alone, and they interact with each other to form networks or pathways so as to carry out biological functions [7], [8], [9], [10], [11]. Therefore, it is crucial to reveal the essential biological mechanisms from a system perspective, and pathway-based analysis is becoming a popular method of analyzing high-throughput data. Several approaches have been proposed to score known pathways by the coherency of expression changes among their member genes [12], [13], [14], [15], [16]. Generally, a known pathway is drawn from sources such as the Gene Ontology (GO) [17] and KEGG [18] databases. In contrast to the documented pathways, however, it is a more difficult task to identify novel sub-networks or pathways responsive to phenotypes from biomolecular networks. Recently, gene-set-based or pathway-based analysis has been extended to perform classification of microarray data by exploiting the phenotype difference [19], [20], [21] and a number of approaches have been demonstrated for not scoring known pathways but extracting relevant sub-networks based on coherent expression patterns of the corresponding genes in the protein-protein interaction (PPI) networks [22], [23], [24], [25]. However, these approaches are mainly molecule-complex-based [23], [25] or individual-gene-based analysis, such as [19], in which the authors indicated that candidate sub-networks are seeded with a single protein and iteratively expanded to add other proteins into the sub-networks. Note that, in biology, a complex is a cluster of genes or proteins so closely related that they intergrade [26], while a pathway is a group of genes or proteins that are interacted (or related) [1].

In contrast to existing works, in this paper we first developed a novel module-based method to identify phenotype-based responsive modules by integrating gene expression data and high-quality PPI networks, which are able to reveal the potential causal or dependent relations between network modules and biological phenotypes. Specifically, we formulated the problem to identify phenotype-based responsive modules as a multi-classification problem of modules on phenotypes by a mathematical programming model, rather than identifying individual genes and gene sets, where the modules are resulted from the topological structure of the PPI networks. Then, the proposed method was applied to the cell-cycle process of budding yeast *Saccharomyces cerevisiae* (*S. cerevisiae*) to identify phenotype-based responsive modules and further examine their regulation roles in the phase transition process based on the microarray data of our biological experiments.

The cell cycle process, by which one cell grows and divides into two daughter cells, is a vital biological process, the regulation of which is highly conserved among the eukaryotes [27], [28]. Although extensive studies have been conducted on the cell cycle process [29], [30], in particular by modeling the budding yeast [31], [32], [33], many detail regulations still remain unclear from network viewpoint. Generally, there are mainly two ways to perturb a biological system, that is, external stimulus, such as exposure to DNA-damaging agents, methyl methanesulfonate (MMS) [34], [35], [36], and internal stimulus, such as knocking out some genes [34], [37]. To functionally relate network modules to different phenotypes, we designed biological experiments by combining these two types of stimuli so as to create various phenotypes for the cell cycle process. In our biological experiments, when adding MMS at 15 min (G1 phase) or knocking out *elg1* at the beginning of the cell cycle process, the cell cycle continues, nevertheless, when adding MMS at 15 min (G1 phase) to *elg1* mutant strains, the cell cycle arrests at S phase.

By the proposed module-based method with exploiting high-throughput data of various phenotypes resulted from our biological experiments, we identified phenotype-based responsive modules and dynamical transition modules of budding yeast cell cycle. A responsive module means that the module potentially plays an important role in some phenotypes, while a transition module indicates that the module is potentially responsible for the transition from one phenotype to another from a dynamical perspective. After the identification of phenotype-based and transition-based responsive modules for the cell cycle phases and their transitions, the identified responsive modules were also validated by classifying the cell cycle phases of two independent datasets on budding yeast cell cycle.

Based on the computational and experimental results, the main contributions of this work can be summarized as follows. First, our method is able to identify phenotype-based and transition-based responsive network modules, drawn from the topological structure of a biomolecular network, instead of dissecting complexes or individual gene-based pathways. Second, the identified modules lead to new insights into the cell cycle process and provide biological interpretations on the functional roles of network modules. In particular, according to the validation on the other two independent datasets and also the functional validation, the phenotype-based responsive modules are potentially signatures or network biomarkers of the cell cycle process. We revealed the reason of arresting cell cycle at S phase under both internal and external stimuli from a network viewpoint, that is, we identified one well-known module “*CLN1 CLN2 CLN3 BUD2*” involved in cell cycle process and two new modules “*PKC1 TOS2 KEL2 PPZ2 SKN7*” and “*SSD1 LST8 TOR1 KOG1 TOR2*”, whose dysfunction results in cell cycle arresting. Third, our method is also a new theoretical model for multi-classification analysis, which was used to study the cell cycle process by relating network modules to different phenotypes and even phase transitions from a dynamical perspective. In addition, we showed that the identified responsive modules can also be directly used to annotate functions of genes or proteins.

## Methods

### Microarray Experiment

**Figure 1:**
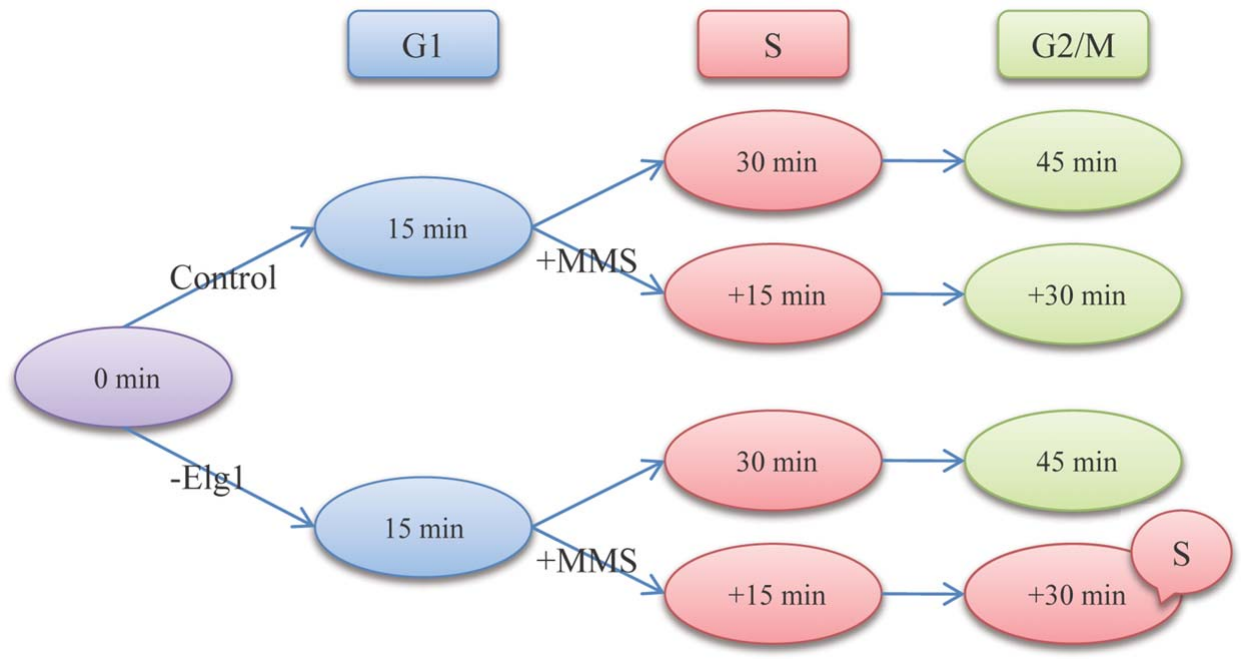
Schematic illustration of our experiment design. The cell cycle is decomposed into three phases, G1, S and G2/M, corresponding to time points of 15 min, 30 min and 45 min, respectively. In the figure, “0 min” means the starting point of cell cycle. “Control” implies WT, while “-*elg1*” implies *elg1* mutant. “+MMS” refers to adding MMS to yeast strains at 15 min, and “+15 min” and “+30 min” signify after 15 min and 30 min’s MMS exposure, respectively. Note that when knocking out *elg1* and then adding MMS at 15 min, cell cycle arrests at S phase. The experiment has two biological repeats for each condition.

The biological experiment was performed on *S. cerevisiae*. SH521 strain was used as the wild-type source (WT). Since *elg1* involves in DNA replication and genome integrity, we knocked out *elg1* gene of SH521 using PCR based one-step gene replacement [38], [39]. WT and *elg1* mutant were cultured in rich YPD medium (1% yeast extract, 2% peptone, and 2% glucose) at 30°C as described [40]. Cells were synchronized to early G1 phase by adding 20 ng/ml alpha factor (US Biology) as described [41], and exposed to 0.01% DNA-damage reagent methyl methanesulfate (MMS, Sigma) after being released into pre-warmed YPD medium. Cells at the indicated time points shown in Figure 1 were harvested for flow cytometry analysis [42] and total RNA isolation [43]. RNA samples were subjected to gene-microarray analysis with Affymetrix GeneChip® Yeast Genome S98 arrays and Scanner GeneChip® 3000. Two biological replicates have done for each condition. The microarray data are available at http://www.aporc.org/doc/wiki/CellCycle.

### Data Preprocessing

The microarray data of our experiment contains 5714 genes and 20 samples. The CEL files were preprocessed by RMA algorithm in R bioconductor package (www.bioconductor.org). Probe sets were mapped to NCBI Entrez gene symbols using the Affymetrix annotation. If there are multiple probe sets corresponding to the same gene, we average them individually. First, because of the fact that many genes of microarray data were irrelevant or redundant [44], we applied ANOVA method to identify genes that were differentially expressed on one or more of the ten groups relative to the others (p-value <0.05). As a result, 4443 genes from microarray data were finally retained for further analysis. Second, we downloaded a curated PPI network of *S.* *cerevisiae* from MIPS [45], and we only considered those PPIs, of which both interacted proteins are contained in our microarray data.

### Identifying Responsive Modules by a Mathematical Programming Model

**Figure 2:**
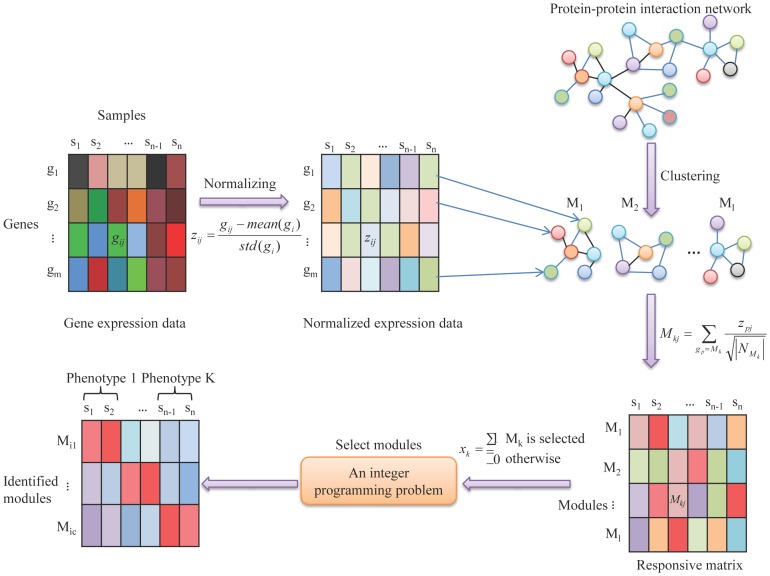
Schematic flowchart of the proposed method. First, gene expression values were normalized over all samples, and PPI network was decomposed into modules by clustering algorithm. Then a responsive value can be defined for each module by combining the z-scores of genes in the corresponding module, i.e., the responsive matrix is formed. To select responsive modules for different phenotypes, we defined a variable 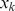 representing whether a module is selected, and further formulated this problem by an integer programming model. Finally, we identified the responsive modules by solving the integer programming problem to classify the phenotypes.

Figure 2 illustrates the schematic flowchart of our method. On the one hand, given the microarray data with 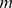 genes and 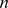 samples, gene expression values 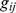 were normalized to *z*-transformed scores 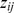, which for each gene 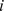 has mean 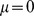 and deviation 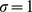 over all samples 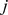. On the other hand, we decomposed the PPI network into 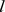 modules by the Markov Clustering (MCL) algorithm [46]. To integrate the gene microarray data and PPI modules, we mapped the normalized expression values of each gene on its corresponding protein in the modules, and then we defined the module responsive value as a combined z-score [19] for 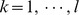 and 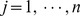,
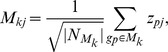
where 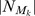 is the number of proteins in the module 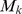. In this way, we obtained the responsive matrix with element 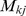 representing the responsive value of module 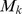 in the case of sample 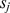. To identify responsive modules under different phenotypes or conditions, we defined 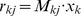, where 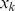 is 1 if module 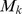 is selected, otherwise 0. Therefore, nonzero 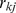 means that module 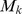 is selected, while we do not select module 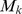 if 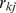 is zero.

Assume that there are 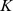 phenotypes among 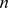 samples, to relate 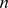 samples to 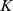 phenotypes, we aimed to classify the 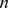 samples into 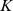 clusters. Meanwhile, we intended to identify the minimum number of responsive modules in this multi-classification process. In this study, we exploited the idea of K-means clustering to formulate such a multi-classification problem as a mathematical programming model, which aims to minimize the within-cluster error sum of squares, that is,
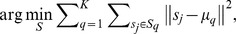
where 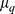 is the mean of sample points 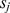 in the sample cluster 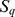. Based on this framework, we calculated the values of 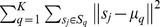 for all possible classifications, and denoted these values as 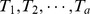, respectively (Note that 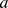 in 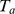 corresponds to all the classifications). On the other hand, for the targeted classification, the value is written as 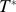. Hence, if all conditions 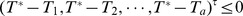
are satisfied, we have successfully classified the samples, where superscript 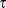 means the transpose of a vector or a matrix. By expanding 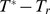, which is the function of 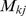 and 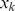, we can further express the conditions as



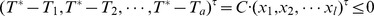
(see Supporting Information S1 for more details), where 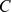
is a matrix function of 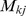 with element 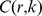 representing the k-th module’s contribution to the *r*-th condition. From the above analysis, we formulated the module-identification problem as the following binary integer programming:



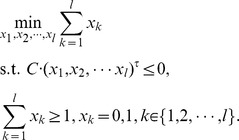



### Algorithm of Solving Binary Integer Programming Problem

Clearly, the formulated integer programming problem is NP-hard. Therefore, we have to adopt other techniques to obtain an approximate solution due to the computational complexity of this problem.

In the integer programming problem, we aimed to identify the number of modules as small as possible but with high accuracy of the classification. Therefore, we defined an index to evaluate the effect of classification, that is, the average power of classification of modules. For the purpose, an approximate algorithm is designed as follows:

Step 1). Rank the modules according to their scores, 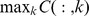, increasingly, which evaluate the power of classification of the *k*-th module individually, where 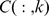 means the *k-*th column of matrix 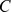.

Step 2). Add the module one by one to compute the average power of classification of modules,
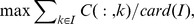
where 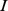 is the set of chosen modules and 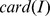 is the cardinality of set 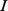, according to the ranking in Step 1). When the minimum value of 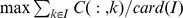 is achieved, we select the ahead 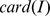 number of modules in the ranking in Step 1) as the responsive modules.

## Results

### Sample Clustering

The microarray data of our biological experiment totally contains 5714 genes and 20 samples. The first batch of 10 samples are composed of 5 control samples and 5 *elg1* mutant samples (see Figure 1), and the other batch of 10 samples are biological replicate of the first batch. There are totally ten phenotypes in our experiment and we will identify responsive modules to these phenotypes as well as their transitions. We extracted 4443 genes with differential information underlying these phenotypes by ANOVA (see Methods). To group these samples from a global respective, we implemented the hierarchical clustering on these genes and the results are shown in Supporting Information S2. We found that the 20 samples are almost successfully classified into ten classes of phenotypes, that is, the biological replicates and three time points of cell cycle phases are clustered together. The various phenotypes of our experiment are correlated with the genome-wide gene expression profiles. Therefore, we focus our subsequent analyses on these 4443 genes for identifying responsive and transition modules corresponding to these phenotypes.

### Responsive Modules of Cell Cycle Phases for Each Group of Conditions

**Figure 3:**
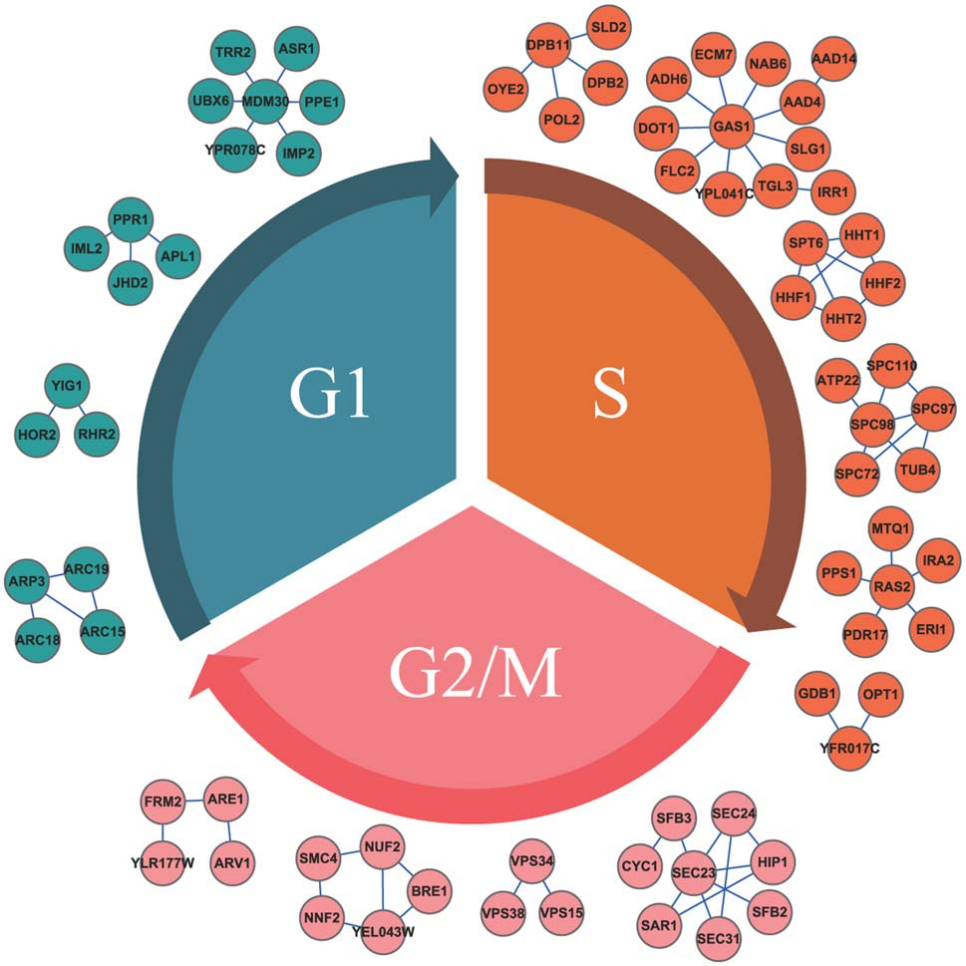
The identified responsive modules in the control group. The responsive modules for different cell cycle phases in the control group are shown. The modules are arranged on the phase in which their maximum responsive values are achieved. The colors of blue, yellow and pink correspond to cell cycle phases, G1, S and G2/M, respectively.

For our experiments, we categorized the 20 samples into four groups, i.e., control group, MMS group, *elg1* mutant group, and *elg1* mutant MMS group. By implementing our method, we identified 14, 17, 8 and 23 responsive modules for the control group, MMS group, *elg1* mutant group and *elg1* mutant MMS group, respectively (see Supporting Information S2). In the control group, we arranged the responsive modules on the corresponding cell cycle phases, at which the maximum responsive values of the modules are achieved. The results of 14 responsive modules of three cell cycle phases are shown in Figure 3. The three cell cycle phases can be discriminated by these responsive modules according to our method. In our integer programming model, they are the minimal number of modules with the maximum average discrimination power for distinguishing the different phenotypes. The discrimination power underlying these modules indicates that they are subnetwork biomarkers of reflecting the progression status during the cell cycle process.

In Figure 3, for instance, as a network-based biomarker for S phase, in which the DNA is synthesized and chromosomes replicated, the identified responsive module “*POL2 DPB11 DPB2 SLD2 OYE2*” contains the genes performing these kinds of functions, which are consistent with the biological functions of S phase. Specifically, *POL2* is a catalytic subunit of DNA polymerase (II) epsilon, a chromosomal DNA replication polymerase that exhibits processivity and proofreading exonuclease activity, and also involved in DNA synthesis during DNA repair [47]. *DPB11* is a replication initiation protein that loads DNA pol epsilon onto pre-replication complexes at origins and a checkpoint sensor recruited to stalled replication forks by the checkpoint clamp complex where it activates Mec1p [48]. *DPB2* is the second largest subunit of DNA polymerase (II) epsilon, and is required for normal yeast chromosomal replication, whose expression peaks at the G1/S phase boundary [49]. *SLD2* is a protein required for DNA replication, and is phosphorylated at S phase by S-phase cyclin-dependent kinases (CDKs) [50]. As a member of the module, it implicates that *OYE2* may be also involved in the S phase. Moreover, the identified module “*NUF2 NNF2 SMC4 BRE1 YEL043W*” possesses the consistent function in G2/M phase, in which the chromosomes are separated and the cell is divided into two daughters. *NUF2* is involved in chromosome segregation, spindle checkpoint activity and kinetochore clustering [51]. *NNF2* plays a role in chromosome segregation [52]. *SMC4* reorganizes chromosomes during cell division [53]. *BRE1* is found to be required for double-strand break repair (DSBR), transcription, silencing, and checkpoint control [54].

Furthermore, as an illustration for other groups, the module “*SIF2 PIB2 HOS4*” in *elg1* mutant MMS group is involved in the negative regulation of meiosis [55], which is consistent with the phenotype of *elg1* mutant MMS group. In this module, *SIF2* is a WD40 repeat-containing subunit of the *SET3C* histone deacetylase complex, which represses early/middle sporulation genes and antagonizes telomeric silencing [56], *PIB2* is a protein binding phosphatidylinositol 3-phosphate, involved in telomere-proximal repression of gene expression [57], and *HOS4* is a subunit of the *SET3* complex, which is a meiotic-specific repressor of sporulation specific genes that contains deacetylase activity [56].

From the above analyses, we concluded that the identified responsive modules indeed characterize the corresponding phenotypes, which confirms the effectivenss of our method. Each module can be regarded as a network-based biomarker for the cell cycle process, which is not individual-gene-based or molecule-complex-based, but rather a functional-module-based signature decomposed from the PPI network. This feature is quite different from conventional pathway-based analysis, which is still based on individual genes, and then extended to subnetworks. To some extent, the identified discriminative phenotype-based responsive modules reflect the endogenous dynamics of the cell cycle, which also indicate that the different responses and similar drivers correspond to various phenotypes in the progression of development phases.

### Transition Modules between Different Phases of Cell Cycle

**Figure 4:**
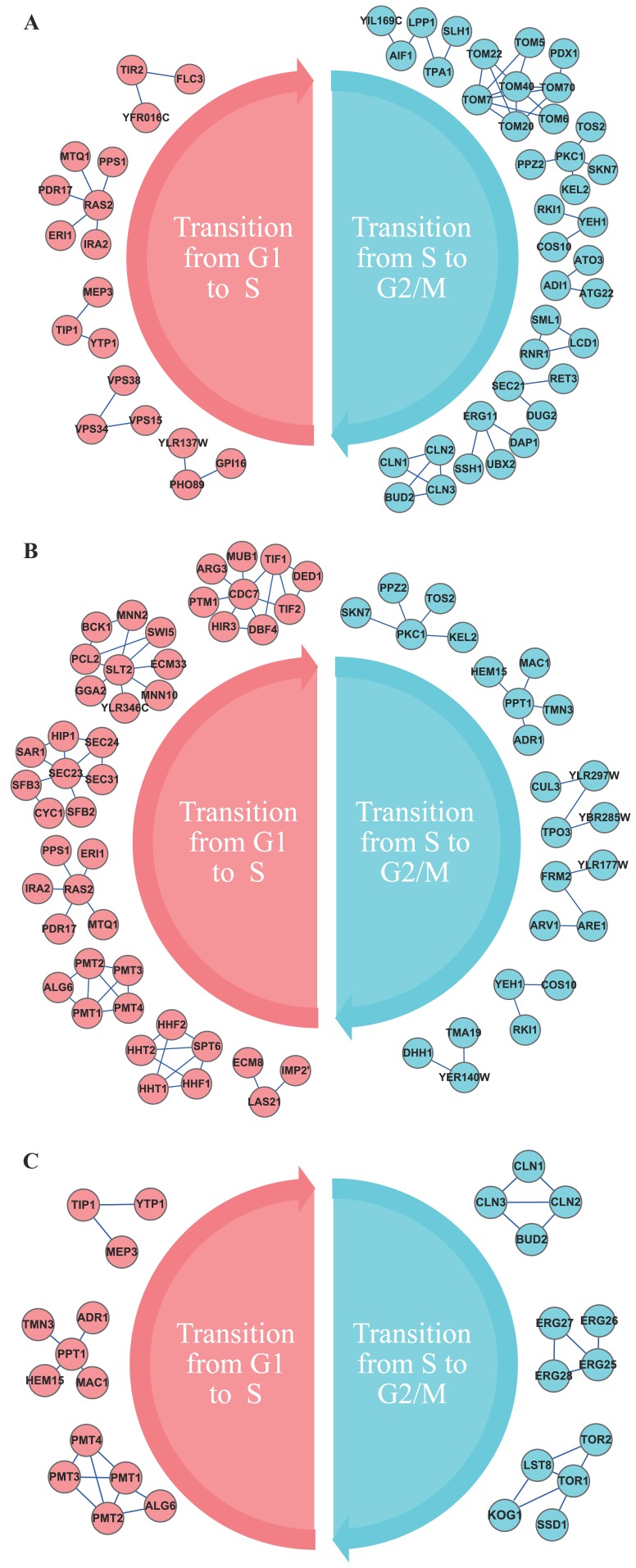
The identified transition modules. Part of the transition modules from G1 phase to S phase, and from S phase to G2/M phase under external stimulus (A), internal stimulus (B) and both stimuli (C) are presented. The pink corresponds to the transition from G1 phase to S phase, while the deep green indicates the transition from S phase to G2/M phase.

The transition modules refer to those modules that are potentially responsible for the dynamical transition of phases in the cell-cycle process. We identified these modules by our method based on the classification of the dynamics and these phenotypes. Compared to the control condition, we identified these transition modules from G1 phase to S phase and from S phase to G2/M phase under internal stimulus, external stimulus and both, respectively. The procedure of identifying the transition modules from G1 phase to S phase under external stimulus is performed by classifying the samples of G1 phase and S phase under external stimulus and the control condition. The similar procedures were also implemented for other phase transitions in other groups. The identified transition modules between these phases are shown in Figure 4. The significant differences between these modules in these phases indicate that they are biological signatures and changeover markers for the cell-cycle phase transition.

By relating network modules to phenotypes shown in Table 1, we noted that two modules “*PKC1 TOS2 KEL2 PPZ2 SKN7*” and “*RKI1 COS10 YEH1*”, are responsive for the transition from S phase (30 min) to G2/M phase (45 min) under internal stimulus (Figure 4A) or external stimulus (Figure 4B), but they are not identified under both stimuli (Figure 4C). Furthermore, we also found three specific transition modules in Table 1 for phase arresting under both stimuli (Figure 4C), that is, “*SSD1 LST8 TOR1 KOG1 TOR2*”, “*ERG26 ERG25 ERG28 ERG27*” and “*CLN1 CLN2 CLN3 BUD2*”.

**Table 1:** Relations between transition modules and phenotypes.

30 min – 45 min transition modules				
phenotype	Transition (from S to G2/M phase)		Arresting (at S phase)	
Transition modules	Nodes	Edges	Nodes	Edges
	RKI1 COS10 YEH1	COS10 YEH1 RKI1 YEH1	SSD1 LST8 TOR1 KOG1 TOR2	TOR1 LST8 TOR2 LST8 TOR1 TOR2 SSD1 TOR1 KOG1 LST8 KOG1 TOR1
	PKC1 TOS2 KEL2 PPZ2 SKN7	PKC1 TOS2 PKC1 KEL2 PKC1PPZ2 PKC1 SKN7	ERG26 ERG25 ERG28 ERG27	ERG25 ERG26 ERG25 ERG27 ERG25 ERG28 ERG27 ERG28
			CLN1 CLN2 CLN3 BUD2	CLN3 CLN2 BUD2 CLN2 CLN3 CLN1 CLN1 CLN2 CLN3 BUD2

Transition modules for the transition from S to G2/M phase: common transition modules from S to G2/M phase under conditions adding MMS and knocking out *elg1*.

In the module “*PKC1 TOS2 KEL2 PPZ2 SKN7*”, *PKC1* is a protein serine/threonine kinase essential for cell wall remodeling during growth [58], *TOS2* is a protein involved in localization of *CDC24p* to the site of bud growth [59], *KEL2* is a protein that functions in a complex with *KEL1p* to negatively regulate mitotic exit [60], *PPZ2* is a serine/threonine protein phosphatase Z, and is involved in regulation of potassium transport, which affects cell cycle progression [61]. The analysis on the functional module of marking the transition concludes that the cooperation of these genes in the subnetwork plays an important role for the dynamical transition from S phase to G2/M phase.

Furthermore, identification of the transition modules provides more evidence of the differences of cell-cycle-phase phenotypes and indicates the causal units for the cell development, especially in the phase-transition points. In our experiment, we paid a special attention to the identified transition modules shown in Figure 4C, which correspond to the transition from S phase (30 min) to S phase (45 min). With the aim of identifying the functional modules of critical transition and development of these phases, our experiment generated various phenotypes by stimuli. When we implemented the internal stimulus of gene mutant and external stimulus of DNA damage, the cell cycle would arrest at the S phase. With this in mind, the identified modules potentially play essential roles in the process of cell cycle arresting at S phase caused by the double stimuli. In the modules, “*CLN1 CLN2 CLN3 BUD2*” and “*SSD1 LST8 TOR1 KOG1 TOR2*”, *CLN1*, *CLN2* and *CLN3* are well-known cyclins involved in regulation of the cell cycle [62], and BUD2 is a GTPase activating factor for *RSR1p*/*BUD1p* required for both axial and bipolar budding patterns [63]. Moreover, *TOR1* and *TOR2* are two PIK-related protein kinases and constitute a complex, which is involved in meiosis, *SSD1* is a translational repressor and cooperates with *Tor* complex to maintain cellular integrity, and *LST8* and *KOG1* are known to play roles in *TOR* signaling pathway [64].

From the above analysis of these transition modules (Table 1), we concluded that it is the dysfunctions of these modules that possibly contribute to cell cycle arresting at S phase responsive to gene mutant of *elg1*and DNA damage of MMS. The details of the transition modules under different stimuli are shown in Supporting Information S1 and S2.

### Functional Analysis of Responsive Modules

We detected various responsive modules for the multiple phenotypes of cell cycles individually. A part of these functional distinct modules are identified among a series of processes contributing to cell cycle, development and DNA integrity in budding yeast. The enriched functions of these identified modules include DNA replication, DNA repair, checkpoint signaling, chromosome segregation and cell division (see Table 2). The results of GO functional enrichment analysis are shown in Supporting Information S2.

**Table 2:** List of modules that are related to a series of processes of cell cycle, such as DNA replication, DNA repair, checkpoint signaling, chromosome segregation, and cell division.

Module	Term ID	Size	Node	Edge	P-value	Description
SSD1 LST8 TOR1 KOG1 TOR2	GO: 0001558	13	5	6	6.99e-11	Regulation of cell growth
SPC110 SPC97 TUB4 SPC98SPC72 ATP22	GO: 0005819	86	6	8	3.42e-09	Spindle
SEC31 SEC23 SAR1 HIP1 SEC24SFB3 CYC1 SFB2	GO: 0048193	187	8	10	2.52e-08	Golgi vesicle transport
SIS2 SIT4 SAP155 HIS4 SAP185	GO: 0000082	51	5	7	2.43e-08	G1/S transition of mitotic cell cycle
POL2 OYE2 DPB11 DPB2 SLD2	GO: 0006298	17	5	4	8.42e-07	Mismatch repair
ARC18 ARC15 ARP3 ARC19	GO: 0005856	231	4	4	2.26e-06	Cytoskeleton
SIF2 PIB2 HOS4	GO: 0045835	13	3	2	1.35e-05	Negative regulation of meiosis
LCD1 RNR1 SML1	GO: 0006260	160	3	3	1.97e-05	DNA replication
CDC7 DBF4 TIF1 ARG3 TIF2 HIR3MUB1 PTM1 DED1	GO: 0001100	6	9	12	3.11e-05	Negative regulation of exit from mitosis
MSH5 SWE1 HSL7 AIM10	GO: 0000086	35	4	3	1.02e-04	G2/M transition of mitotic cell cycle
NUF2 NNF2 BRE1 SMC4 YEL043W	GO: 0007059	131	5	6	1.03e-04	Chromosome segregation
DUG2 SEC21 RET3	GO: 0005798	42	3	2	1.48e-04	Golgi-associated vesicle
CLN1 CLN2 CLN3 BUD2	KEGG: 04111	125	4	5	5.56e-04	Cell cycle
ERD2 SED1 SED4 HEM13	GO: 0006888	89	4	3	1.33-03	ER to Golgi vesiclemediated Transport
PKC1 TOS2 KEL2 PPZ2 SKN7	GO: 0007346	88	5	4	3.19e-03	Regulation of mitotic cell cycle
BUD14 REF2 TVP18 ZPS1	GO: 0000903	8	4	3	5.40e-03	Cell morphogenesis during vegetative growth
WHI4 PCL6 YER156C	GO: 0000307	11	3	2	5.57e-03	Cyclin-dependent protein kinase holoenzyme complex
TPA1 LPP1 AIF1 SLH1	GO: 0042981	6	5	4	5.09e-03	Regulation of apoptosis
APM2 GAL10 YFP045W	GO: 0030140	13	3	2	6.58e-03	Trans-Golgi network transport vesicle
YIR016W MOB2 YOL036W	GO: 0007096	24	3	2	8.10e-03	Regulation of exit from mitosis
SPT6 HHF1 HHF2 HHT1 HHT2	GO: 0006333	51	5	8	8.62e-03	Chromatin assembly or disassembly

“Size” refers to the number of genes or proteins in the corresponding “Term ID”, “Node” and “Edge” indicate the number of nodes and edges in the corresponding modules, and “P-value” is the statistical significance of the random overlapped nodes between “module” and “Term ID” by the hypergeometric test.

For instance, as a responsive module in both MMS group and *elg1* mutant MMS group, module “*MSH5 SWE1 HSL7 AIM10*” is involved in mitotic cell cycle process [55], where *MSH5* is a protein of the *MUTS* family which forms a dimer with *MSH4p* that facilitates crossovers between homologs during meiosis [65]. *SWE1* is a protein kinase that regulates the G2/M transition by inhibition of *CDC28p* kinase activity [66]. And *HSL7* is a protein arginine N-methyltransferase that exhibits septin and *HSL1p*-dependent bud neck localization as well as periodic *HSL1p*-dependent phosphorylation. It is required along with *HSL1p* for bud neck recruitment, phosphorylation, and degradation of *SWE1p* [67], [68]. *AIM10*, whose biological process is still unclear (http://www.yeastgenome.org/), is a protein with similarity to tRNA synthetases [69]. From the functional analysis of this module, we concluded that *AIM10* is related to the cell cycle process.

The functional analysis of these modules indicates that they are the biomarkers for the phenotypes. Specially, the identified transition modules clearly offer a potential clue to explain why the cell cycle arrests at S phase. It is the dysfunction of the modules “*PKC1 TOS2 KEL2 PPZ2 SKN7*”, “*CLN1 CLN2 CLN3 BUD2*” and “*SSD1 LST8 TOR1 KOG1 TOR2*” that result in cell cycle arresting. On the other hand, because the functions of components of a module are believed to be related [2], we can attribute some functions of components in one module to those not well known genes, i.e., annotate the functions of those genes. Based on this idea, we concluded that the component *AIM10* of the module “*MSH5 SWE1 HSL7 AIM10*” may be related with mitotic cell cycle, although its biological process is still not known, and the component *OYE2* of the module “*POL2 DPB11 DPB2 SLD2 OYE2*” may be involved in one or more of the processes of DNA replication, DNA repair, and mismatch repair. In addition, we found that the identified modules contain dynamical complexes, such as the module “*CLN1 CLN2 CLN3 BUD2*”, which is consistent with the idea of [23] to some extent, however, the results of our method are more specific and extended in some sense.

### Responsive Modules are Informative of Classifying Cell Cycle Phases

**Figure 5:**
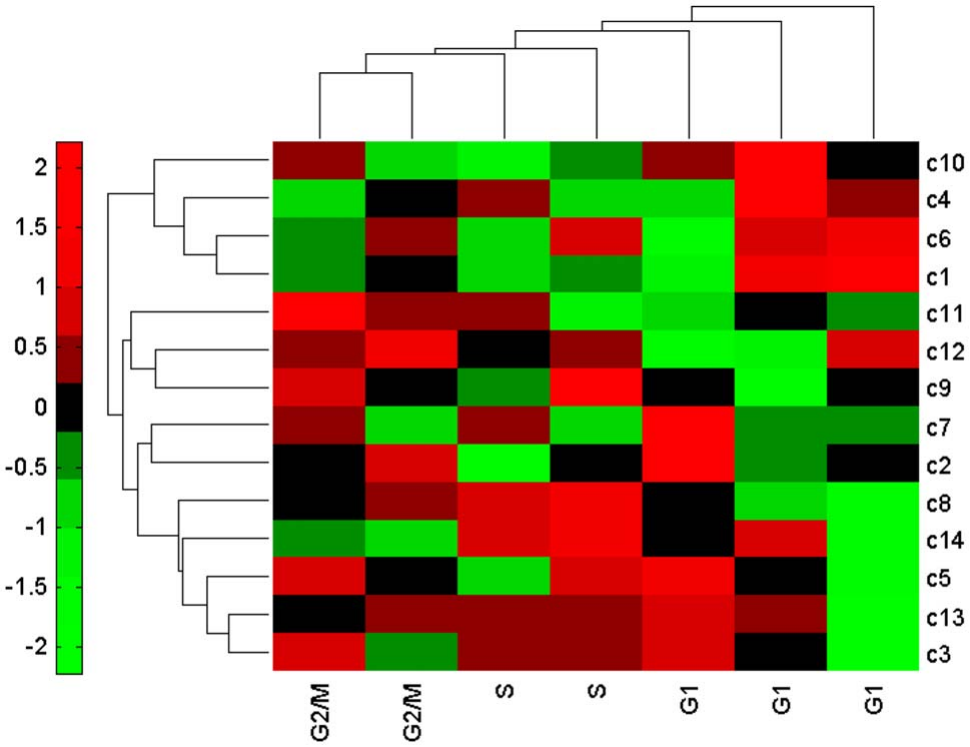
Dendrogram and heat map of the first independent test dataset GSE3635 based on the identified responsive modules. The row labels denote the module IDs in the control group (see Supporting Information S1), while the column labels indicate three cell cycle phases G1, S, G2/M. The color legend represents the responsive value.

**Figure 6:**
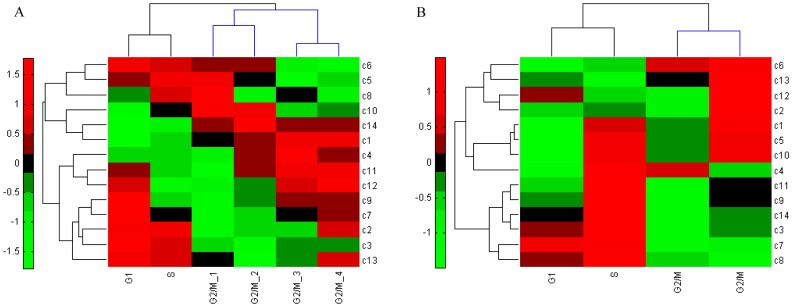
Dendrogram and heat map of the second independent test dataset GSE8799 based on the identified responsive modules. The row labels denote the module IDs in the control group, while the column labels indicate three cell cycle phases G1, S, G2/M in the first cell cycle (A), in the second cell cycle (B). The color legend represents the responsive value.

To validate that the identified responsive modules of various cell-cycle phenotypes are informative and general, we conducted the classification of cell cycle phases by these modules based on two independent datasets. Specifically, we tested the samples of the first cell cycle in the first dataset GSE3635 [29]. And we chose the samples of the first cell cycle in WildType_rep1 in the second dataset GSE8799 [30]. In addition, we also tested the samples of the second cell cycle. The results are shown in Figures 5 and 6, respectively, and we successfully classified the three cell-cycle phases of the two independent datasets. Therefore, we concluded that these responsive modules are capable of correctly identifying their expression features in various cell-cycle phenotypes, and are important clue of marking cell development stages. The validation not only provides evidence on the effectiveness and advantage of our method, but also verifies that the identified modules functionally correspond to the phenotypes of the cell cycle process.

## Discussion

In this paper, by integrating high-throughput gene expression data and PPI network, we developed a novel method to identify responsive modules and dynamical transition modules for various phenotypes and phase transitions under internal and external stimulus of yeast cell cycle process. In this work, to alleviate the problem of noisy data of protein-protein interactions, we chose a widely-used gold standard [70], [71] PPI database for *S. cerevisiae*, i.e., MIPS [45] as our source network for analysis. However, the noise inside may also have influence on the results. Additionally, for yeast PPI, we have checked other databases, such as IntAct [72] and BioGRID [73]. We found that, compared to MIPS, although these two databases contain more interactions, the overlap between MIPS and IntAct (or BioGRID) is relatively small. Therefore, we chose the high-quality PPIs documented in MIPS as our reference network. Our method is network-based or functional module-based analysis, different from the existing works, which are mainly individual-gene-based or molecule-complex-based study [19], [23], [25]. In addition, although we adopted the phenotype difference to identify responsive modules, our method is quite different from those exploited the similar tasks [19], [20], [21]. For instance, in [21], the authors obtained gene sets by classifying biological functions of genes and then identified the gene sets according to their significant difference between two phenotypes, while our method focused on functional modules and formulated the module identification as an integer programming problem.

Our method is based on the ideas of K-means clustering and exploits the supervised clustering to identify responsive and transition modules. In other words, our work focuses on identifying the functional modules (or subnetworks from the network perspective), rather than identifying genes (or gene sets) with similar expression values or patterns under multi-conditions (e.g., biclustering methods [16]). Therefore, the biclustering method and our method are quite different approaches which are designed from two different perspectives.

Based on the experiment, we identified responsive modules for the groups of conditions and transition modules for the dynamical phase transitions in yeast cell-cycle process. As biological signatures or network biomarkers for the cell cycle, functional analyses show that some identified modules are involved in a series of processes such as DNA replication, DNA repair, checkpoint signaling, chromosome segregation, and cell division, which contribute to cell cycle and DNA integrity in yeast. The transition modules characterize a dynamical process from one phenotype to another, and therefore our method offers a new alternative to study dynamical processes of biological systems from the viewpoint of network modules, which leads to new biological insights. In particular, from a dynamical perspective, we showed that modules “*PKC1 TOS2 KEL2 PPZ2 SKN7*”, “*CLN1 CLN2 CLN3 BUD2*” and “*SSD1 LST8 TOR1 KOG1 TOR2*” potentially play an essential role in phase arresting. This explains why the cell cycle arrests at the S phase when adding MMS to *elg1* mutant strains at 15 min. As validation of generality, we tested these identified responsive modules in two independent datasets of the cell cycle process. The results in Figures 5 and 6 indicate that we not only presented the expression change of modules, but also gave the corresponding cell cycle stages from our biological experiments of internal and external stimuli, i.e., phenotypes, thereby verifying the effectiveness of our findings. Further biological experiment to validate the results is our future topic.

Although we restricted our work on integrating microarray gene expression data and PPI network, the method can be straightforwardly extended to other areas, such as multi-classification problem (e.g., disease classification), other type of high-throughput data and biomolecular network analysis. Due to the small sample size, we adopted with-cluster error sum of squares as an optimization function (see Methods), but other simpler classifiers can also be used in a similar manner to identify the responsive modules when the sample size is reasonably large.

In summary, by formulating the identification of phenotype-based responsive modules as a mathematical programming problem, we proposed a general method to identify phenotype-based responsive modules and further revealed possible causal or dependent relations between network modules and biological phenotypes of budding yeast cell cycle. The resulting responsive modules provide new insight into the regulation mechanisms of cell-cycle process from a network viewpoint. Clearly, the identification of transition modules offers a new way to study dynamical processes at a functional module level. In this paper, we have considered that the changes of the module activity under different biological conditions. However, the composition of the modules may also vary under different conditions. Therefore, it is also an interesting research topic for identifying the composition variations in these modules in addition to their activities.

## Supporting Information

Supporting Information S1 (XLS)

Supporting Information S2 (DOC)
