# Supplementary material for: Identifying Responsive Modules by Mathematical Programming: An Application to Budding Yeast Cell Cycle
Source: PLoS One. 2012 Jul 25;7(7):e41854. doi: 10.1371/journal.pone.0041854 (PMC3405030; doi:10.1371/journal.pone.0041854)
Supplement: Supporting Information S2 — (DOC) [file pone.0041854.s002.doc]

Supplementary information for “Identifying phenotype-based responsive modules by mathematical programming: an application to budding yeast cell cycle”

Zhenshu Wen, Zhi-Ping Liu, Yiqing Yan, Guanying Piao, Zhengrong Liu, Jiarui Wu, Luonan Chen

[**Section 1: Supplementary figures 1**](#__RefHeading___Toc295743128)

[**Section 2: Supplementary tables 7**](#__RefHeading___Toc295743129)

[**Section 3: Supplementary text 17**](#__RefHeading___Toc295743130)

## Section 1: Supplementary figures.

**Supplementary Figure S1. Hierarchical clustering diagram of samples based on 4443 genes.**

**Supplementary Figure S2. The number of responsive modules identified in control group, according to the maximum average power of classification.**

**Supplementary Figure S3. The number of responsive modules identified in MMS group, according to the maximum average power of classification.**

**Supplementary Figure S4. The number of responsive modules identified in Elg1 mutant group, according to the maximum average power of classification.**

**Supplementary Figure S5. The number of responsive modules identified in Elg1 mutant MMS group, according to the maximum average power of classification.**

**Supplementary Figure S6. The number of transition modules identified from 15 min (G1 phase) to 30 min (S phase) under external stimulus, according to the maximum average power of classification.**

**Supplementary Figure S7. The number of transition modules identified from 30 min (S phase) to 30 min (G2/M) phase under external stimulus, according to the maximum average power of classification.**

**Supplementary Figure S8. The number of transition modules identified from 15 min (G1 phase) to 30 min (S phase) under internal stimulus, according to the maximum average power of classification.**

**Supplementary Figure S9. The number of transition modules identified from S phase to G2/M phase under internal stimulus, according to the maximum average power of classification.**

**Supplementary Figure S10. The number of transition modules identified from 30 min (G1 phase) to 45 min (S phase) under both internal stimulus and external stimulus, according to the maximum average power of classification.**

**Supplementary Figure S11. The number of transition modules identified from 30 min (S phase) to 45 min (S phase) under both internal stimulus and external stimulus, according to the maximum average power of classification.**

## Section 2: Supplementary tables

**Table S2: Functional Analysis of the identified responsive modules for control group**

| Responsive modules for control group | | | | | | | |
| --- | --- | --- | --- | --- | --- | --- | --- |
| No. | Module | Term ID | size | node | edge | P-value | Description |
| 1 | ARC18 ARC15 ARP3 ARC19 | GO: 0005856 | 231 | 4 | 4 | 2.26e-06 | Cytoskeleton |
| 2 | SEC31 SEC23 SAR1 HIP1 SEC24 SFB3 CYC1 SFB2 | GO: 0048193 | 187 | 8 | 10 | 2.52e-08 | Golgi vesicle transport |
| 3 | SPC110 SPC97 TUB4 SPC98 SPC72 ATP22 | GO: 0005819 | 86 | 6 | 8 | 3.42e-09 | Spindle |
| 4 | MDM30 IMP2 YPR078C UBX6 TRR2 PPE1 ASR1 | GO: 0034599 | 6 | 7 | 6 | 7.08e-03 | Cellular response to oxidative stress |
| 5 | NUF2 NNF2 BRE1 SMC4 YEL043W | GO: 0007059 | 131 | 5 | 6 | 1.03e-04 | Chromosome segregation |
| 6 | PPR1 APL1 JHD2 IML2 | GO: 0016706 | 6 | 4 | 3 | 4.05e-03 | Oxidoreductase activity |
| 7 | AAD14 DOT1 IRR1 GAS1 SLG1 FLC2 TGL3 YPL041C AAD4 NAB6 ADH6 ECM7 | GO: 0071554 | 227 | 12 | 11 | 5.05e-05 | Cell wall organization or biogenesis |
| 8 | ARE1 FRM2 ARV1 YLR177W | GO: 0006629 | 262 | 4 | 3 | 3.32e-04 | Lipid metabolic process |
| 9 | POL2 OYE2 DPB11 DPB2 SLD2 | GO: 0006298 | 27 | 5 | 4 | 8.42e-07 | Mismatch repair |
| 10 | VPS38 VPS15 VPS34 | GO: 0005768 | 112 | 3 | 2 | 6.60e-06 | endosome |
| 11 | ERI1 RAS2 PDR17 MTQ1 IRA2 PPS1 | GO: 0006140 | 7 | 6 | 5 | 1.80e-05 | Regulation of nucleotide metabolic process |
| 12 | YFR017C GDB1 OPT1 | GO: 0015926 | 18 | 3 | 2 | 9.10e-03 | Glucosidase activity |
| 13 | SPT6 HHT2 HHF1 HHT1 HHF2 | GO: 0006333 | 51 | 5 | 8 | 8.62e-03 | Chromatin assembly or disassembly |
| 14 | RHR2 YIG1 HOR2 | KEGG: 00561 | 14 | 3 | 2 | 8.00e-05 | Glycerolipid metabolism |

**Table S3: Functional Analysis of the identified responsive modules for MMS group**

| Responsive modules for MMS group | | | | | | | |
| --- | --- | --- | --- | --- | --- | --- | --- |
| No. | Module | Term ID | size | node | edge | P-value | Description |
| 1 | ALG3 WBP1 ALG5 OST2 STT3 OST1 SWP1 RFM1 RNR2 FYV10 | GO: 0016758 | 83 | 10 | 13 | 9.58e-12 | Transferase activity, transferring hexosyl groups |
| 2 | VPS38 VPS15 VPS34 | GO: 0005768 | 112 | 3 | 2 | 6.60e-06 | endosome |
| 3 | MSH5 SWE1 HSL7 AIM10 | GO: 0000086 | 35 | 4 | 3 | 1.02e-04 | G2/M transition of mitotic cell cycle |
| 4 | ARE1 FRM2 ARV1 YLR177W | GO: 0006629 | 262 | 4 | 3 | 3.32e-04 | Lipid metabolic process |
| 5 | ERG26 ERG25 ERG28 ERG27 | KEGG: 00100 | 15 | 4 | 4 | 7.96e-07 | Steroid biosynthesis |
| 6 | SCO2 COX17 SCO1 | GO: 0005507 | 29 | 3 | 3 | 1.06e-07 | Copper ion binding |
| 7 | RKI1 COS10 YEH1 | GO: 0016860 | 16 | 3 | 2 | 8.09e-03 | Intramolecular oxidoreductase activity |
| 8 | LCB1 SLC1 ESF1 | GO: 0006643 | 29 | 3 | 2 | 6.94e-05 | Membrane lipid metabolic process |
| 9 | SHO1 THO2 KIN2 | GO: 0071214 | 23 | 3 | 2 | 7.76e-03 | Cellular response to abiotic stimulus |
| 10 | GLO4 YRO2 RVB1 | GO: 0033202 | 11 | 3 | 2 | 5.57e-03 | DNA helicase complex |
| 11 | BUD14 REF2 TVP18 ZPS1 | GO: 0000903 | 8 | 4 | 3 | 5.40e-03 | Cell morphogenesis during vegetative growth |
| 12 | YFR045W APM2 GAL10 | GO: 0030140 | 13 | 3 | 2 | 6.58e-03 | Trans-Golgi network transport vesicle |
| 13 | GUS1 OPI1 MTC2 | GO: 0003714 | 15 | 3 | 2 | 5.06e-03 | Transcription corepressor activity |
| 14 | STE2 VPS62 YMR265C | GO: 0000755 | 7 | 3 | 2 | 2.36e-03 | Cytogamy |
| 15 | WHI4 PCL6 YER156C | GO: 0000307 | 11 | 3 | 2 | 5.57e-03 | Cyclin-dependent protein kinase holoenzyme complex |
| 16 | TRM9 ARG81 ARG80 | GO: 0006525 | 17 | 3 | 2 | 2.33e-05 | Arginine metabolic process |
| 17 | RHR2 YIG1 HOR2 | KEGG: 00561 | 14 | 3 | 2 | 8.00e-05 | Glycerolipid metabolism |

**Table S4: Functional Analysis of the identified responsive modules for Elg1 mutant group**

| Responsive modules for Elg1 mutant group | | | | | | | |
| --- | --- | --- | --- | --- | --- | --- | --- |
| No. | Module | Term ID | size | node | edge | P-value | Description |
| 1 | PDX1 TOM70 TOM6 TOM7 TOM20 TOM22 TOM40 TOM5 | GO: 0005742 | 8 | 8 | 14 | 1.27e-21 | Mitochondrial outer membrane translocase complex |
| 2 | CUL3 YBR285W TPO3 YLR297W | GO: 0042787 | 14 | 4 | 3 | 7.08e-03 | Protein ubiquitination during ubiquitin-dependent protein catabolic process |
| 3 | CDC73 VPS71 SLM3 VPS8 QRI5 MDM35 VID30 IST3 BUD13 IES2 MRPS8 RRP6 RTR1 | GO: 0006406 | 66 | 13 | 13 | 8.83e-03 | mRNA export from nucleus |
| 4 | SSC1 MGE1 MDJ1 | GO: 0051082 | 72 | 3 | 3 | 1.73e-06 | Unfolded protein binding |
| 5 | ALG3 WBP1 ALG5 OST2 STT3 OST1 SWP1 RFM1 RNR2 FYV10 | GO: 0016758 | 83 | 10 | 13 | 9.58e-12 | Transferase activity, transferring hexosyl groups |
| 6 | RPA12 YBR220C RSM7 YDL129W PET100 CAB5 GDT1 SDH4 YBR219C | GO: 0008177 | 5 | 9 | 8 | 6.74e-03 | Succinate dehydrogenase (ubiquitin) activity |
| 7 | VIK1 SEC61 SSS1 PIM1 | GO: 0006620 | 8 | 4 | 3 | 9.58e-06 | Posttranslational protein targeting to membrane |
| 8 | PRP40 SNU71 ALR1 FAA3 | GO: 0005685 | 18 | 4 | 3 | 5.22e-05 | U1 snRNP |

**Table S5: Functional Analysis of the identified responsive modules for Elg1 mutant MMS group**

| Responsive modules for Elg1 mutant MMS group | | | | | | | |
| --- | --- | --- | --- | --- | --- | --- | --- |
| No. | Module | Term ID | size | Node | edge | P-value | Description |
| 1 | HSC82 YDJ1 STI1 SSA1 CNS1 HSP82 | GO: 0043248 | 7 | 6 | 9 | 1.80e-05 | Proteasome assembly |
| 2 | OXA1 RPC34 RPC82 RPC31 | GO: 0034062 | 37 | 4 | 4 | 8.96e-07 | RNA polymerase activity |
| 3 | ALG3 WBP1 ALG5 OST2 STT3 OST1 SWP1 RFM1 RNR2 FYV10 | GO: 0016758 | 83 | 10 | 13 | 9.58e-12 | Transferase activity, transferring hexosyl groups |
| 4 | DIE2 OST5 OST3 OST4 | GO: 0006487 | 45 | 4 | 3 | 2.92e-09 | Protein amino acid N-linked glycosylation |
| 5 | SCO2 COX17 SCO1 | GO: 0005507 | 29 | 3 | 3 | 1.06e-07 | Copper ion binding |
| 6 | INO80 STT4 GDS1 | GO: 0000165 | 18 | 3 | 2 | 9.10e-03 | MAPKKK cascade |
| 7 | HSE1 BUL1 PBI2 STP22 VPS27 PAF1 | KEGG: 04144 | 33 | 6 | 5 | 9.55e-06 | Endocytosis |
| 8 | RBG2 SUC2 GIR2 FET4 | GO: 0016580 | 5 | 4 | 3 | 3.38e-03 | Intracellular copper ion transport |
| 9 | SIS2 SIT4 SAP155 HIS4 SAP185 | GO: 0000082 | 51 | 5 | 7 | 2.43e-08 | G1/S transition of mitotic cell cycle |
| 10 | CLN1 CLN2 CLN3 BUD2 | KEGG: 04111 | 125 | 4 | 5 | 5.56e-04 | Cell cycle |
| 11 | AAH1 SRX1 BTN2 YLR225C | GO: 0019239 | 13 | 4 | 3 | 8.76e-03 | Deaminase activity |
| 12 | YIR016W MOB2 YOL036W | GO: 0007096 | 24 | 3 | 2 | 8.10e-03 | Regulation of exit from mitosis |
| 13 | STE5 ZTA1 PMD1 | GO: 0010627 | 3 | 3 | 2 | 1.52e-03 | Regulation of protein kinase cascade |
| 14 | ATG19 LAP4 RSB1 YOR302W RRP7 KIN1 | GO: 0034204 | 7 | 6 | 6 | 7.08e-03 | Lipid translocation |
| 15 | ERD2 SED1 SED4 HEM13 | GO: 0006888 | 89 | 4 | 3 | 1.33-03 | ER to Golgi vesicle-mediated transport |
| 16 | VTI1 PEP12 SYN8 | KEGG: 04130 | 10 | 3 | 3 | 3.10e-06 | SNARE interactions in vesicular budding |
| 17 | TOM71 TFC7 TFC1 COX14 | GO: 0000127 | 10 | 4 | 3 | 5.14e-06 | Transcription factor TFIIIC complex |
| 18 | TPS2 TPS3 PMU1 TSL1 | GO: 0016137 | 8 | 4 | 4 | 6.48e-09 | Glycoside metabolic process |
| 19 | VIK1 SEC61 SSS1 PIM1 | GO: 0006620 | 8 | 4 | 3 | 9.58e-06 | Posttranslational protein targeting to membrane |
| 20 | MSH5 SWE1 HSL7 AIM10 | GO: 0000086 | 35 | 4 | 3 | 1.02e-04 | G2/M transition of mitotic cell cycle |
| 21 | TIM12 MRS2 TIM9 | GO: 0006839 | 70 | 3 | 2 | 1.38e-04 | Mitochondrial transport |
| 22 | ACS2 YLR049C ACS1 | GO: 0016877 | 9 | 3 | 2 | 6.16e-06 | Ligase activity, forming carbon-sulfur bonds |
| 23 | SIF2 PIB2 HOS4 | GO: 0045835 | 13 | 3 | 2 | 1.35e-05 | Negative regulation of meiosis |

**Table S6: Functional Analysis of the transition modules from 15 min to 30 min under external stimulus: adding MMS**

| Transition modules from 15 min to 30 min under external stimulus: adding MMS | | | | | | | |
| --- | --- | --- | --- | --- | --- | --- | --- |
| No. | Module | Term ID | size | node | edge | P-value | Description |
| 1 | ERI1 RAS2 PDR17 MTQ1 IRA2 PPS1 | GO: 0006140 | 7 | 6 | 5 | 1.80e-05 | Regulation of nucleotide metabolic process |
| 2 | GPI16 PHO89 YLR137W | GO:0016255 | 5 | 3 | 2 | 2.55e-03 | Attachment of GPI anchor to protein |
| 3 | TIR2 YFR016C FLC3 | GO:0005199 | 16 | 3 | 2 | 8.13e-03 | Structural constituent of cell wall |
| 4 | TIP1 MEP3 YTP1 | GO: 0008519 | 6 | 3 | 2 | 3.06e-03 | Ammonium transmembrane transporter activity |
| 5 | VPS38 VPS15 VPS34 | GO: 0005768 | 112 | 3 | 2 | 6.60e-06 | endosome |

**Table S7: Functional Analysis of the transition modules from 30 min to 45 min under external stimulus: adding MMS**

| Transition modules from 30 min to 45 min under external stimulus: adding MMS | | | | | | | |
| --- | --- | --- | --- | --- | --- | --- | --- |
| No. | Module | Term ID | size | node | edge | P-value | Description |
| 1 | RKI1 COS10 YEH1 | GO: 0016860 | 16 | 3 | 2 | 8.09e-03 | Intramolecular oxidoreductase activity |
| 2 | YIL169C TPA1 LPP1 AIF1 SLH1 | GO: 0042981 | 6 | 5 | 4 | 5.09e-03 | Regulation of apoptosis |
| 3 | CLN1 CLN2 CLN3 BUD2 | KEGG: 04111 | 125 | 4 | 5 | 5.56e-04 | Cell cycle |
| 4 | PDX1 TOM70 TOM6 TOM7 TOM20 TOM22 TOM40 TOM5 | GO: 0005742 | 8 | 8 | 14 | 1.27e-21 | Mitochondrial outer membrane translocase complex |
| 5 | ERI1 RAS2 PDR17 MTQ1 IRA2 PPS1 | GO: 0006140 | 7 | 6 | 5 | 1.80e-05 | Regulation of nucleotide metabolic process |
| 6 | INO80 STT4 GDS1 | GO: 0000165 | 18 | 3 | 2 | 9.10e-03 | MAPKKK cascade |
| 7 | MTF1 PUT4 ALY2 RRT12 | GO: 0031160 | 14 | 4 | 3 | 9.48e-03 | Spore wall |
| 8 | ACS2 YLR049C ACS1 | GO: 0016877 | 9 | 3 | 2 | 6.16e-06 | Ligase activity, forming carbon-sulfur bonds |
| 9 | DUG2 SEC21 RET3 | GO: 0005798 | 42 | 3 | 2 | 1.48e-04 | Golgi-associated vesicle |
| 10 | NUP100 YBP1 YAP1 | GO: 0006979 | 88 | 3 | 2 | 6.57e-04 | Response to oxidative stress |
| 11 | YUR1 RRD2 RRD1 PEX15 | GO: 0019211 | 4 | 4 | 3 | 2.08e-06 | Phosphatase activator activity |
| 12 | YLR143W NSE5 YGK3 | GO: 0030915 | 8 | 3 | 2 | 4.07e-03 | Smc5-smc6 complex |
| 13 | KRE1 VPS28 KEX1 | GO: 0008236 | 18 | 3 | 2 | 9.15e-03 | Serine-type peptidase activity |
| 14 | YGL010W YPL257W ALD5 | KEGG: 00903 | 5 | 3 | 2 | 2.93e-03 | Limonene and pinene degradation |
| 15 | LCD1 RNR1 SML1 | GO: 0006260 | 160 | 3 | 3 | 1.97e-05 | DNA replication |
| 16 | YGL230C PNS1 KTI12 | GO: 0003682 | 80 | 3 | 2 | 4.02e-02 | Chromatin binding |
| 17 | PKC1 TOS2 KEL2 PPZ2 SKN7 | GO: 0007346 | 88 | 5 | 4 | 3.19e-03 | Regulation of mitotic cell cycle |
| 18 | ADI1 ATO3 ATG22 | GO: 0003333 | 4 | 3 | 2 | 2.04e-03 | Amino acid transmembrane transport |
| 19 | VPS38 VPS15 VPS34 | GO: 0005768 | 112 | 3 | 2 | 6.60e-06 | endosome |
| 20 | ERG11 UBX2 DAP1 SSH1 | GO: 0016128 | 28 | 4 | 3 | 1.30e-04 | Phytosteroid metabolic process |

**Table S8: Functional Analysis of the transition modules from 15 min to 30 min under internal stimulus: knocking out Elg1**

| Transition modules from 15 min to 30 min under internal stimulus: knocking out Elg1 | | | | | | | |
| --- | --- | --- | --- | --- | --- | --- | --- |
| No. | Module | Term ID | size | Node | edge | P-value | Description |
| 1 | LSM6 GNT1 YMR221C | GO: 0016684 | 17 | 3 | 2 | 8.59e-03 | Oxidoreductase activity |
| 2 | PMT2 ALG6 PMT1 PMT4 PMT3 | GO: 0006493 | 15 | 5 | 8 | 1.33e-10 | Protein amino acid N-linked glycosylation |
| 3 | SLT2 ECM33 MNN10 PCL2 BCK1 YLR346C SWI5 MNN2 GGA2 | GO: 0000917 | 7 | 9 | 10 | 4.35e-05 | Barrier septum formation |
| 4 | CUL3 TPO3 YBR285W YLR297W | GO: 0042787 | 14 | 4 | 3 | 7.08e-03 | Protein ubiquitination during ubiquitin-dependent protein catabolic process |
| 5 | SPT6 HHT2 HHF1 HHT1 HHF2 | GO: 0006333 | 51 | 5 | 8 | 8.62e-03 | Chromatin assembly or disassembly |
| 6 | TIP1 MEP3 YTP1 | GO: 0008519 | 6 | 3 | 2 | 3.06e-03 | Ammonium transmembrane transporter activity |
| 7 | ALG3 WBP1 ALG5 OST2 STT3 OST1 SWP1 RFM1 RNR2 FYV10 | GO: 0016758 | 83 | 10 | 13 | 9.58e-12 | Transferase activity, transferring hexosyl groups |
| 8 | CDC7 DBF4 TIF1 ARG3 TIF2 HIR3 MUB1 PTM1 DED1 | GO: 0001100 | 6 | 9 | 12 | 3.11e-05 | Negative regulation of exit from mitosis |
| 9 | NUP100 YBP1 YAP1 | GO: 0006979 | 88 | 3 | 2 | 6.57e-04 | Response to oxidative stress |
| 10 | SSA4 CIT3 ALG2 DOS2 | GO: 0004376 | 11 | 4 | 3 | 7.46e-03 | Glycolipid mannosyltransferase activity |
| 11 | CSN9 IKI1 YJR015W | GO: 0008180 | 6 | 3 | 2 | 3.06e-03 | signalosome |
| 12 | ERI1 RAS2 PDR17 MTQ1 IRA2 PPS1 | GO: 0006140 | 7 | 6 | 5 | 1.80e-05 | Regulation of nucleotide metabolic process |
| 13 | UTP15 FMN1 UTP5 | GO: 0006356 | 8 | 3 | 2 | 4.79e-06 | Regulation of transcription from RNA polymerase I promoter |
| 14 | PPR1 APL1 JHD2 IML2 | GO: 0016706 | 6 | 4 | 3 | 4.05e-03 | Oxidoreductase activity |
| 15 | LAS21 ECM8 IMP2' | GO: 0016780 | 14 | 3 | 2 | 7.12e-03 | Phosphotransferase activity, for other substituted phosphate groups |
| 16 | GLK1 EMI2 SHE3 ARG2 FIG1 | GO: 0019200 | 18 | 5 | 4 | 8.69e-05 | Carbohydrate kinase activity |
| 17 | INO80 STT4 GDS1 | GO: 0000165 | 18 | 3 | 2 | 9.10e-03 | MAPKKK cascade |
| 18 | NUP42 BAG7 HAM1 | GO: 0047429 | 5 | 3 | 2 | 2.55e-03 | Nucleoside-triphosphate diphosphatase activity |
| 19 | YOL083W RHB1 AMS1 | KEGG: 00511 | 1 | 3 | 2 | 6.63e-04 | Other glycan degradation |
| 20 | PKC1 TOS2 KEL2 PPZ2 SKN7 | GO: 0007346 | 88 | 5 | 4 | 3.19e-03 | Regulation of mitotic cell cycle |
| 21 | SFG1 YAE1 IZH1 | GO: 0006882 | 10 | 3 | 2 | 5.06e-03 | Cellular zinc ion homestasis |
| 22 | MAC1 PPT1 HEM15 ADR1 TMN3 | GO: 0003700 | 137 | 5 | 4 | 5.83e-03 | Sequence-specific DNA binding transcription factor activity |
| 23 | YAF9 LAC1 LAG1 LIP1 | KEGG: 00600 | 13 | 4 | 4 | 6.86e-05 | Sphingolipid metabolism |
| 24 | VPS38 VPS15 VPS34 | GO: 0005768 | 112 | 3 | 2 | 6.60e-06 | endosome |
| 25 | CTM1 YGR071C NOP9 | GO: 0016278 | 16 | 3 | 2 | 6.58e-03 | Lysine N-methyltransferase activity |
| 26 | RKI1 COS10 YEH1 | GO: 0016860 | 16 | 3 | 2 | 8.09e-03 | Intramolecular oxidoreductase activity |
| 27 | NRP1 YBL081W NGR1 | GO: 0010494 | 19 | 3 | 2 | 2.96e-05 | Stress granule |
| 28 | KRE1 VPS28 KEX1 | GO: 0008236 | 18 | 3 | 2 | 9.15e-03 | Serine-type peptidase activity |
| 29 | SYH1 COS2 YPR063C | GO: 0000322 | 182 | 3 | 2 | 8.89e-02 | Storage vacuole |
| 30 | MPD1 ASA1 PDI1 EUG1 | GO: 0016860 | 16 | 4 | 3 | 1.62e-08 | Intramolecular oxidoreducase activity |
| 31 | STE5 ZTA1 PMD1 | GO: 0010627 | 3 | 3 | 2 | 1.52e-03 | Regulation of protein kinase cascade |
| 32 | SEC31 SEC23 SAR1 HIP1 SEC24 SFB3 CYC1 SFB2 | GO: 0048193 | 187 | 8 | 10 | 2.52e-08 | Golgi vesicle transport |
| 33 | YGR035C RTC5 CRS5 | GO: 0010035 | 38 | 3 | 2 | 6.42e-03 | Response to inorganic substance |
| 34 | YIL169C TPA1 LPP1 AIF1 SLH1 | GO: 0042981 | 6 | 5 | 4 | 5.09e-03 | Regulation of apoptosis |

**Table S9: Functional Analysis of the transition modules from 30 min to 45 min under internal stimulus: knocking out Elg1**

| Transition modules from 30 min to 45 min under internal stimulus: knocking out Elg1 | | | | | | | |
| --- | --- | --- | --- | --- | --- | --- | --- |
| No. | Module | Term ID | size | Node | edge | P-value | Description |
| 1 | CUL3 TPO3 YBR285W YLR297W | GO: 0042787 | 14 | 4 | 3 | 7.08e-03 | Protein ubiquitination during ubiquitin-dependent protein catabolic process |
| 2 | PKC1 TOS2 KEL2 PPZ2 SKN7 | GO: 0007346 | 88 | 5 | 4 | 3.19e-03 | Regulation of mitotic cell cycle |
| 3 | TMA19 DHH1 YER140W | GO: 0000932 | 13 | 3 | 2 | 6.58e-03 | Cytoplasmic mRNA processing body |
| 4 | ARE1 FRM2 ARV1 YLR177W | GO: 0006629 | 262 | 4 | 3 | 3.32e-04 | Lipid metabolic process |
| 5 | MAC1 PPT1 HEM15 ADR1 TMN3 | GO: 0003700 | 137 | 5 | 4 | 5.83e-03 | Sequence-specific DNA binding transcription factor activity |
| 6 | RKI1 COS10 YEH1 | GO: 0016860 | 16 | 3 | 2 | 8.09e-03 | Intramolecular oxidoreductase activity |

**Table S10: Functional Analysis of the transition modules from 15 min to 30 min under both internal and external stimulus: knocking out Elg1 and adding MMS**

| Transition modules from 15 min to 30 min under both internal and external stimulus: knocking out Elg1 and adding MMS | | | | | | | |
| --- | --- | --- | --- | --- | --- | --- | --- |
| No. | Module | Term ID | size | Node | edge | P-value | Description |
| 1 | PMT2 ALG6 PMT1 PMT4 PMT3 | GO: 0006493 | 15 | 5 | 8 | 1.33e-10 | Protein amino acid N-linked glycosylation |
| 2 | TIP1 MEP3 YTP1 | GO: 0008519 | 6 | 3 | 2 | 3.06e-03 | Ammonium transmembrane transporter activity |
| 3 | MAC1 PPT1 HEM15 ADR1 TMN3 | GO: 0003700 | 137 | 5 | 4 | 5.83e-03 | Sequence-specific DNA binding transcription factor activity |

**Table S11: Functional Analysis of the transition modules from 30 min to 45 min under both internal and external stimulus: knocking out Elg1 and adding MMS**

| Transition modules from 30 min to 45 min under both internal and external stimulus: knocking out Elg1 and adding MMS | | | | | | | |
| --- | --- | --- | --- | --- | --- | --- | --- |
| No. | Module | Term ID | size | Node | edge | P-value | Description |
| 1 | SSD1 LST8 TOR1 KOG1 TOR2 | GO: 0001558 | 13 | 5 | 6 | 6.99e-11 | Regulation of cell growth |
| 2 | ERG26 ERG25 ERG28 ERG27 | KEGG: 00100 | 15 | 4 | 4 | 7.96e-07 | Steroid biosynthesis |
| 3 | CLN1 CLN2 CLN3 BUD2 | KEGG: 04111 | 125 | 4 | 5 | 5.56e-04 | Cell cycle |

## Section 3: Supplementary text

**Text S1: The constricted conditions of the binary integer programming**

The value of the within-cluster sum of squares is calculated as follows:

$$T=\sum_{q=1}^{K} \sum_{j=1}^{n_{q}} \left\|s_{j}-\frac{s_{1}+\cdots+s_{n_{q}}}{n_{q}}\right\|^{2}$$

$$=\sum_{q=1}^{K} \sum_{j=1}^{n_{q}} \left\|(r_{1j},\cdots,r_{lj})^{\tau}-\frac{(r_{11},\cdots,r_{l1})^{\tau}+\cdots+(r_{1n_{q}},\cdots,r_{l n_{q}})^{\tau}}{n_{q}}\right\|^{2}$$

$$=\sum_{q=1}^{K} \sum_{j=1}^{n_{q}} \left\|(M_{1j}x_{1},\cdots,M_{lj}x_{l})^{\tau}-\frac{(M_{11}x_{1},\cdots,M_{l1}x_{l})^{\tau}+\cdots+(M_{1n_{q}}x_{1},\cdots,M_{l n_{q}}x_{l})^{\tau}}{n_{q}}\right\|^{2}$$

$$=\sum_{q=1}^{K} \sum_{j=1}^{n_{q}} \left\|((M_{1j}-\frac{M_{11}+\cdots+M_{1n_{q}}}{n_{q}})x_{1},\cdots,(M_{lj}-\frac{M_{l1}+\cdots+M_{l n_{q}}}{n_{q}})x_{l})^{\tau}\right\|^{2}$$

$$=\sum_{q=1}^{K} \sum_{j=1}^{n_{q}} [(M_{1j}-\frac{M_{11}+\cdots+M_{1n_{q}}}{n_{q}})^{2}x_{1}^{2}+\cdots+(M_{lj}-\frac{M_{l1}+\cdots+M_{l n_{q}}}{n_{q}})^{2}x_{l}^{2}]$$

$$=\sum_{q=1}^{K} \sum_{j=1}^{n_{q}} (M_{1j}-\frac{M_{11}+\cdots+M_{1n_{q}}}{n_{q}})^{2}x_{1}^{2}+\cdots+\sum_{q=1}^{K} \sum_{j=1}^{n_{q}} (M_{li}-\frac{M_{l1}+\cdots+M_{l n_{q}}}{n_{q}})^{2}x_{l}^{2}$$

$$=\sum_{q=1}^{K} \sum_{j=1}^{n_{q}} (M_{1j}-\frac{M_{11}+\cdots+M_{1n_{q}}}{n_{q}})^{2}x_{1}+\cdots+\sum_{q=1}^{K} \sum_{j=1}^{n_{q}} (M_{lj}-\frac{M_{l1}+\cdots+M_{l n_{q}}}{n_{q}})^{2}x_{l}$$

$$=(\sum_{q=1}^{K} \sum_{j=1}^{n_{q}} (M_{1j}-\frac{M_{11}+\cdots+M_{1n_{q}}}{n_{q}})^{2},\cdots,\sum_{q=1}^{K} \sum_{j=1}^{n_{q}} (M_{lj}-\frac{M_{l1}+\cdots+M_{l n_{q}}}{n_{q}})^{2})\cdot(x_{1},\cdots,x_{l})^{\tau}$$

Therefore, obviously,

$$\begin{matrix} T^{*}-T_{r}=(\sum_{q=1}^{K} \sum_{j=1}^{n_{q}} (M_{1j}-\frac{M_{11}+\cdots+M_{1n_{q}}}{n_{q}})^{2}-\sum_{q=1}^{K_{r}} \sum_{j=1}^{n_{q}} (M_{1j}-\frac{M_{11}+\cdots+M_{1n_{q}}}{n_{q}})^{2},\cdots, \\ \sum_{q=1}^{K} \sum_{j=1}^{n_{q}} (M_{lj}-\frac{M_{l1}+\cdots+M_{l n_{q}}}{n_{q}})^{2}-\sum_{q=1}^{K_{r}} \sum_{j=1}^{n_{q}} (M_{lj}-\frac{M_{l1}+\cdots+M_{l n_{q}}}{n_{q}})^{2})\cdot(x_{1},\cdots,x_{l})^{\tau} \end{matrix}$$

$\mathop{=}\limits^{\Delta} C(r,:)\cdot(x_{1},\cdots,x_{l})^{\tau}$ for $r=1,2,\cdots,a$.

It follows that,

$(T^{*}-T_{1},T^{*}-T_{2},\cdots,T^{*}-T_{a})^{\tau}=C\cdot(x_{1},x_{2},\cdots x_{n})^{\tau}$ □
